# Bioactive Properties and Phenolic Composition of Wood-Aged Beers: Influence of Oak Origin and the Use of Pale and Dark Malts

**DOI:** 10.3390/foods12061237

**Published:** 2023-03-14

**Authors:** Julio C. Machado, Pedro D. M. Nicola, Olga Viegas, Mickael C. Santos, Miguel A. Faria, Isabel M. P. L. V. O. Ferreira

**Affiliations:** 1 LAQV/REQUIMTE, Laboratory of Bromatology and Hydrology, Department of Chemical Sciences, Universidade do Porto, R. Jorge Viterbo Ferreira 228, 4050-313 Porto, Portugal; 2 Faculty of Nutrition and Food Sciences, Universidade do Porto, 4150-180 Porto, Portugal; 3 J. Dias Cooperage, Rua da Lomba 513, 4500-526 Espinho, Portugal

**Keywords:** beer maturation, wood chips, HiVan, MTT, FRAP

## Abstract

Ageing beer in contact with wood is a common technological procedure that has been used for centuries to improve colour, structure, and certain flavours. Herein, the impact of the addition of French and American oak wood to two beer styles, pale and dark, on beer phenolic composition (total phenolics, total flavonoids, and HPLC-DAD) and bioactivity (FRAP, DPPH, anti-inflammatory activity in RAW 264.7, and antiproliferative in Caco-2 cells) was assessed. Thirteen phenolics were quantified with values according to previous reports. Dark malt resulted in higher values of total phenolics, to which m-hydroxybenzoic, syringic, p-coumaric acids, and xanthohumol contributed considerably; the exception was (+)-catechin and salicylic acid, which were found to be higher in pale beers. American oak significantly increased 3,4-dihydroxyphenylacetic, vanillic, and syringic acids up to roughly 3, 2, and 10 times, respectively, when compared with French wood. FRAP and DPPH values varied between pale and dark beers, with a less pronounced effect after wood addition. All samples presented considerable cellular antioxidant and anti-inflammatory as well as antiproliferative activity, but differences were found only for the antiproliferative activity, which was higher for the dark beers, which reached about 70% inhibition. Overall, the influence of malts was more pronounced than that of wood, in the studied conditions, highlighting the overwhelming impact of malts on the bioactivity of beer.

## 1. Introduction

Beer is a fermented alcoholic beverage traditionally brewed using water, malted barley, hops, and yeast. It is one of the most popular drinks all over the world, and its consumption keeps trending due to its relatively low cost and potential health benefits when consumed in moderation. The brewing process comprises several stages, and although some can vary, depending on the specific beer type/style, others are always used. The fundamental stages are malting, the production of wort (mashing, lautering, boiling, clarification), fermentation, and downstream processing such as maturation in cold, filtration, and the ageing processes [1,2].

Ageing beer in contact with wood is a common technological procedure that has been used for centuries to improve colour, structure, and certain flavours. Wood impacts beer due to physical–chemical reactions taking place during the contact process, which include the extraction of volatile and phenolic compounds, decomposition and esterification of the wood lignins, and, in the case of maturation in barrels, the evaporation of volatile compounds and micro-oxygenation, which improves oxidation, polymerisation, and condensation reactions [3]. This technological process has begun to shift from the traditional practice of immersing the beverage in wood (e.g., storage and maturation in barrels) to the other way around: adding wood to the beer in the form of chips, cubes, and spirals, which greatly increases the extractable surface area of the wood. This last process thus enables more efficient extraction of compounds, diminishing the lignins, evaporation, and micro-oxygenation effects [4]. In addition to the improvement of sensory characteristics, the extractable phenolic compounds from woods contribute to an increase in the antioxidant capacity of beers [5], leading to an increment in bioactivity configuring a change in the paradigm of wood-aged processes in alcoholic beverage production. The reported effects of bioactivity increasing after maturation in wood have been described in other beverages such as wine [4].

The extraction of phenolic compounds and, consequently, increased antioxidant capacity [6] depend on many factors such as wood type, origin, degree of toasting, and format. The most popular type of wood selected for this purpose is oak (*Quercus* L.) from different origins, such as France or America, which is reported to possess significant amounts of phenolic compounds [7], mainly gallic acid, sinapaldehyde, protocatechuic acid, ellagic acid, coniferaldehyde, vanillin, caffeic acid, syringaldehyde, vanillic, ferulic, and p-coumaric acids. American oak woods are obtained from *Q. alba*, and French oaks are obtained from *Q. robur* or *Q. petraea*. The toast degree of the wood is also crucial for the amounts of individual phenolics, as different compounds are impacted differently by the toasting process (an increase in protocatechuic aldehyde, vanillin, syringaldehyde, coniferaldehyde, and sinapaldehyde and some phenolic acids such as vanillic and ferulic) [7].

The present work aimed, thus, to assess the effects of the wood-ageing process on beer bioactivity and phenolic composition. For that purpose, two types of beers (produced form pale and dark malts) and wood chips from two origins (USA and France) were tested via the addition of wood in the maturation stage of the brewing process. From a bioactivity standpoint, biochemical analysis regarding phenolic content—total phenolics (TFC), total flavonoids (TFC), Ferric Reducing Antioxidant Power (FRAP), and 2,2-diphenyl-1-picryl-hydrazyl-hydrate (DPPH) assays—and in vitro bioactivity in human cells—antiproliferative, antioxidant, and anti-inflammatory activities—were carried out to better understand the potential enrichment process. Furthermore, the most abundant phenolic compounds were quantified in beer samples through HPLC-DAD analysis.

## 2. Materials and Methods

### 2.1. Chemical and Materials

The beer ingredients consisting of pale ale (Bamberg, Germany), chocolate, and black malt (Heidelberg, Germany), hops from the American Centennial variety, and the yeast Fermentis SafAle S-04 (Marcq-en-Barœul, France) were acquired at a local brew store. Oak wood chips from the HiVan toast type (OakWise, Winchester, VA, USA) were kindly provided by the cooperage J. Dias & Cª, SA (Espinho, Portugal).

Phenolics analysis was performed via HPLC-DAD with the solvents (HPLC grade) acetonitrile (ACN), ethyl acetate, and methanol (MeOH) obtained from Honeywell, Riedel-de-Haën (Seetze, Germany), whereas acetic acid (LC-MS grade) was obtained from Biosolve Chimie (Dieuze, France). Ultrapure water of 0.055 µS.cm^−1^ was obtained through a Seralpur Pro 90CN system (Seral, Ransbach-Baumbach, Germany). The standards (−)-epicatechin; (+)-catechin; 3,4-dihydroxybenzyl alcohol; 3,4-dihydroxyphenylacetic acid; 4-hydroxycoumarin; caffeic acid; carvacrol; chlorogenic acid; ellagic acid; gallic acid; gentisic acid; kaempferol; m-hydroxybenzoic acid; myricetin; naringenin; naringin; p-coumaric acid; p-hydroxybenzoic acid; protocatechuic acid; quercetin; salicylic acid; sinapic acid; syringic acid; trans-cinnamic acid; trans-ferulic acid; vanillic acid; and vanillin were purchased from Sigma-Aldrich Corp. (St. Louis, MO, USA). Isoxanthohumol, syringaldehyde, and xanthohumol were from Extrasynthese (Lyon, France). For bioactivity assays, aluminium chloride hexahydrate; 2,2-diphenyl-1-picrylhydrazyl; gallic acid (≥98% purity); iron (III) chloride; iron (II) sulphate heptahydrate; sodium nitrite; 2,3,5-Triphenyltetrazolium chloride; and Trolox (97% purity) were acquired from Sigma-Aldrich Corp. The Folin–Ciocalteu reagent (F-C) was purchased from Merck (Darmstadt, Germany). The human colorectal adenocarcinoma Caco-2 cell line (passages 82–87) was supplied by the “Molecular Physical-Chemistry” Research Group of the University of Coimbra, Portugal, whereas the murine macrophage RAW 264.7 cell line (passages 6–7) was obtained from the American Type Culture Collection (LGC Standards S.L.U., Barcelona, Spain). High glucose Dulbecco’s modified Eagle’s medium (DMEM), minimum essential medium non-essential amino acids (MEM NEAA) 100×, GlutaMAXTM 10×, fetal bovine serum (FBS), 0.25% Trypsin-EDTA solution, and Penicillin/Streptomycin 10× solution (10,000 Units·mL^−1^/10,000 µg·mL^−1^) were all purchased from Gibco (Paisley, United Kingdom). 5(6)-Carboxy-2′,7′-dichlorofluorescein diacetate (DCF-DA); dimethyl sulfoxide; the lipopolysaccharide (LPS) from *Escherichia coli* O111:B4; MTT (3-(4,5-dimethylthiazol-2-yl)-2,5-diphenyltetrazolium bromide); N-[naphth-1-yl]ethylenediamine dihydrochloride (NED); phosphate-buffered saline (PBS); and the sulphanilamide were acquired from Sigma-Aldrich Corp. For the fluorescence assays, 96-well black plates with a clear bottom were obtained from Greiner Bio-One (Frickenhausen, Germany). The 0.22 µm nylon syringe filters were purchased from CHM (CHMLAB GROUP, Terrassa-Barcelona, Spain).

### 2.2. Brewing Process

The light beer was produced from 5 kg of pale ale (5.5–7.5 EBC) malt, and the dark one from 4.5 kg pale ale + 0.4 kg chocolate (800–1000 EBC) + 0.1 kg of black (1100–1200 EBC) malt; such malt profiles are widely used in pale ale and stout beer styles, respectively. The brewing process took place in a 30 L electric kettle (Klarstein, Berlin). In the mashing step, the grist was added to the boiler and frequently revolved for 60 min at 65 °C. At the end of the mashing step, the absence of starch was assessed using an iodine solution, the temperature was raised to approximately 78 °C for enzymatic inactivation, and the wort was obtained via filtration through the grain and boiled for 60 min. Hops were added at the 30 min mark. The wort was cooled, transferred to a 30 L fermenter, and yeast-inoculated (SafAle^TM^ S-04, Fermentis, Marquette-lez-Lille, France). Fermentation was performed for 7 days at 20 °C, and maturation was performed for 5 days at 4 °C. Beer was then aliquoted in 1.5 L bottles and aged with French and American oak wood chips at 2 g·L^−1^ for 21 days at 22 °C, according to supplier instructions. Chips were processed with considerations for their uniformity to increase the reproducibility of the wood addition to replicates. Six different samples (Table 1) were obtained, aliquoted in 50 mL tubes, and stored at −20 °C until analysis. Samples destined for the cell culture assays were immediately frozen at −80 °C and lyophilised for ethanol removal.

### 2.3. Phenolic Composition of Beers

#### 2.3.1. Total Phenolic (TPC) and Flavonoids (TFC) Contents

TPC was performed using the Folin–Ciocalteu (F-C) method [8]. The assay was carried out in 96-well plates according to the conditions previously described by Magalhães et al. [9]. Briefly, 25 µL of F-C reagent was mixed with 75 µL of ultrapure water and 75 µL of either gallic acid standard solution or diluted samples (1:10-320). The microplate was then incubated for 10 min. Then, 100 μL of NaCO3 was added, and the absorbance at 765 nm was measured after 120 min. TFC was performed via the methodology originally described by Zhishen et al. [10] and was assessed according to the method described by Herald et al. [11]. Samples (200 µL) or standards ((+)-catechin solution) were extracted with 800 µL methanol for 5 min, with agitation, in 2 mL microtubes; the samples were subsequently centrifuged at 5000× *g* for 2 min. The methanolic extract (250 µL) was mixed with 75 µL of a 50 g/L NaNO_2_ solution and 150 µL of a 100 g/L AlCl_3_ solution. Then, after vigorous shaking for 6 min, 500 µL of 1 M NaOH and 500 µL of ultrapure water were added. Finally, the tubes were centrifuged for 5 min at 3220× *g*, and their absorbance was read at 510 nm against a reagent blank. 

#### 2.3.2. Phenolic Quantification via Reverse-Phase High-Performance Liquid Chromatography with Diode Array Detector (HPLC-DAD) Analysis

A salting-out assisted liquid–liquid extraction (SALLE) procedure was performed according to Zhao et al. [12] with some adjustments. For each treatment, beer was degassed through intensive stirring and sonication for a combined time of 30 min. Then, 5 g of NaCl was added to 12.5 mL of the degassed beer sample. The resulting sample was extracted three times by using 12.5 mL of ethyl acetate and centrifuging the sample (10,000× *g*, 10 min) between extractions to assist phase separation. The ethyl acetate extracts were then concentrated using a nitrogen sample evaporator. Finally, the extract was redissolved in MeOH and filtered through a 0.45 µm PTFE membrane.

HPLC-DAD was performed to establish the phenolic compound profile of the tested beer samples. The separation was performed with an AcclaimTM PolarAdvantage C16 (3 µm, 3 × 150 mm, 120 Å) column (ThermoFisher Scientific, MA, USA) with the aid of a SecurityGuard ULTRA C18 guard column and a KrudKatcher ULTRA HPLC In-Line Filter (Phenomenex inc., CA, USA). The Chromeleon 7 chromatography data system (ThermoFisher Scientific, MA, USA) was used for data processing.

Regarding mobile phase composition, solvent A (0.1% acetic acid in water) and solvent B (0.1% acetic acid in acetonitrile) were selected based on the work of Zhao et al. [12]. Chromatographic conditions were optimised to separate the thirty selected phenolic compounds, which was achieved with the following gradient: 0 min, 3% B; 8 min, 3% B; 30 min, 35% B; 37 min, 75% B; 40 min, 75% B; 45 min, 3% B; 50 min, 3% B. Runtime was 50 min, the solvent flow rate was 0.4 mL·min^−1^, and the injection volume was 5 µL. Regarding the detection of the phenolic compounds, four wavelengths (260, 280, 320, and 360 nm) were selected based on Szwajgier et al. [13] to encompass the hydroxybenzoic and hydroxycinnamic acid derivatives and the flavonoid compounds. Compounds were quantified according to an external standard calibration curve. Calibration plots and respective parameters were determined according to the method used by [14], and limits of detection (LOD) and quantification (LOQ) were calculated for the standards based on the standard deviation of y-residuals from the regression line (Sy/x).

### 2.4. Bioactivity Assays

#### 2.4.1. Antioxidant and Anti-Inflammatory Activities

The antioxidant potential of samples was evaluated via free-radical-scavenging activities (FRAP and DPPH) accordingly to several authors [15,16,17]. In addition, cellular antioxidant activity was evaluated via the determinations of the levels of reactive oxygen species (ROS) and nitric oxide (NO) in RAW 264.7 murine macrophages. Cells were seeded in 96-well black microplates at a density of 200,000 cells mL^−1^ and incubated for 24 h in complete medium (CM) composed of DMEM with 10% heat-inactivated foetal bovine serum, 100 U·mL^−1^ penicillin, and 100 mg·mL^−1^ streptomycin under 5% CO_2_ at 37 °C. Afterwards, the medium was removed, and samples were added at a concentration of 12.5% beer in CM (this concentration warrants over 90% of cell viability assessed via the MTT assay). After 2 h, cells were stimulated with 1 µg·mL^−1^ LPS for 22 h. Nitric oxide was then measured in 100 µL of culture medium transferred to a clear-bottom 96-well microplate and an equal volume of Griess reagent (1% sulphanilamide and 0.1% NED in 2% H_3_PO_4_). After 10 min of incubation, absorbance at 560 nm was measured in the dark at room temperature. To access ROS levels, CM was removed, and cells were washed twice with 100 µL of PBS and stained using 100 µL of 10 µM of DCF-DA solution. After a 30 min incubation period (37 °C, in the dark), the DCF-DA solution was aspirated, and the wells were washed twice with 100 µL of PBS. Finally, 100 µL of PBS was added, and the fluorescence was read immediately (λexc: 485 nm/λem: 535 nm). Both ROS and NO production were expressed as a percentage of the LPS-stimulated cells. Both absorbance and fluorescence were read using a BioTek Synergy HT reader (Santa Clara, CA, USA). The basal pro-inflammatory effect induced by the beer samples (without the LPS stimulation) was subtracted from values corresponding to cells treated with beer and LPS stimulated.

#### 2.4.2. Antiproliferative Activity

For the antiproliferative assays, human colorectal adenocarcinoma (Caco-2) cells were seeded in 96-well plates at a density of 1.25 × 10^4^ cells·mL^−1^ in 200 µL CM composed of DMEM supplemented with 10% heat-inactivated foetal bovine serum, 1% GlutaMAX solution, 1% nonessential AA solution, 100 U·mL^−1^ penicillin, and 100 mg·mL^−1^ streptomycin. After cell adhesion for 12 h, the medium was replaced with the test solutions containing serial dilutions of the beer matrixes and incubated at 37 °C with 5% CO_2_ for 48 h. Beer samples were prepared from lyophilised samples reconstituted in CM, centrifuged at 5000× *g* for 10 min, filtered through a 0.22 µm nylon filter, and, subsequently, diluted in CM (concentrations ranging from 0.78 to 100% of beer in CM). A preliminary assay was performed to estimate the IC_50_ value. Afterwards, samples were tested at concentrations near the estimated IC_50_. The MTT assay was then carried out to evaluate the cell viability against a negative control.

### 2.5. Statistical Analysis

All measurements were performed in triplicate and at least two independent experiments in the case of cellular assays. Dependent variables were tested for the distribution of the residuals with the Shapiro–Wilk test. For each variable, if data from all groups (beers) presented normal distribution, statistically significant differences between the means were evaluated via one-way ANOVA using multiple pairwise comparisons. Tukey’s or Welch’s test, due to homogeneity of variances, was or was not, respectively, confirmed via Levene’s test. If at least one beer presented data without normal distribution, the Kruskal–Wallis chi-squared test was performed to determine statistically significant differences between the medians by using the Dunn test for multiple pairwise comparisons. Principal Component Analysis (PCA) was performed using polyphenol composition data as active variables and bioactive activities as supplementary variables (not influencing the component factors’ definition). In analyses, Pearson correlations were used to test the association between the active and supplementary variables. Pearson correlation coefficients indicate strong negative (r ≤ 0.80) and positive (r ≥ 0.80) correlations. ANOVA and Pearson correlation analyses were performed using GraphPad Prism version 6.00 for Windows (GraphPad Software, La Jolla, CA, USA). PCA was performed using the FactoMineR package in R (R Project for Statistical Computing) version 4.1.2. For the Caco-2 cell line assays, the IC_50_ values were determined via curve fitting by using the equation log (inhibitor) vs. response-variable slope (four parameters). Statistical analysis was set for a *p* < 0.05 or *p* < 0.01 significance level.

## 3. Results

### 3.1. Phenolic Composition 

HPLC analysis enabled the quantification of thirteen phenolic compounds out of the 30 analysed in all samples (Table 2). Overall, compared to pale beers, beers containing dark malts presented higher values of total phenolic content (TPC and TFC), to which m-hydroxybenzoic, syringic, p-coumaric acids, and xanthohumol contributed considerably. On the contrary, the concentrations of (+)-catechin and salicylic acid were significantly higher in the pale beer samples.

Beers treated with American oak presented either higher (3,4-dihydroxyphenylacetic, vanillic, and syringic acids in the pale ales; and 3,4-dihydroxyphenylacetic and syringic in the stouts) or similar amounts when compared with the same beer treated with French wood; thus, these were the greatest increases noticed for the pale beer. Beers treated with French wood (PFW and SFW) did not present significant differences, even in comparison with control beers of the same style without wood addition (P and S, respectively), except for syringic acid quantified in PFW and not detected in P and (−)-epicatechin and trans-ferulic acid, which diminished in PFW.

### 3.2. Chemical Antioxidant Activity Measured via FRAP and DPPH Assays

**Figure 1:**
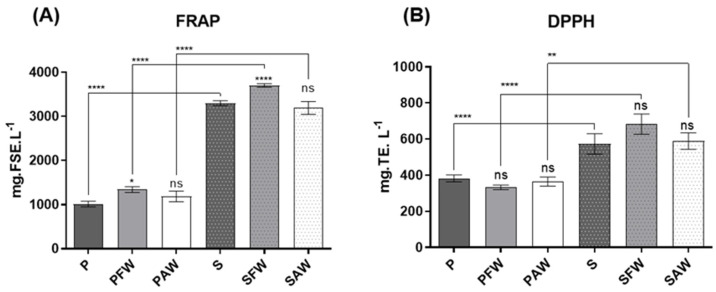
Antioxidant activity assessment through the FRAP (**A**)—expressed in mg of ferrous sulphate equivalents per litre of beer sample (mg FSE·L^−1^), and DPPH (**B**)—assays presented in mg of Trolox equivalents per litre of beer sample (mg TE·L^−1^). Significant differences are indicated over the bars (compared to the respective P and S controls) and over the brackets (comparing the same treatments with different woods). **** Significant at *p* < 0.0001, ** significant at *p* < 0.01, and * significant at *p* < 0.05. ns: not significant. Values expressed as mean ± SEM (n = 3).

Results concerning the chemical antioxidant activities are presented in Figure 1A. FRAP values, expressed in mg of ferrous sulphate equivalents per litre (mg FSE·L^−1^) of sample. Dark beers exhibited a higher antioxidant activity, which varied between 3190.87 ± 145.76 and 3701.50 ± 38.58 mg FSE·L^−1^, when compared to pale ones, which varied between 1014.54 ± 64.59 and 1342.56 ± 66.51 mg FSE·L^−1^. The highest activity of dark beers was independent of the use of wood, highlighting the overwhelming impact of malts on the antioxidant effects of the beverage.

Regarding the wood origin, the beers treated with French oak wood chips (PFW and SFW) presented statistical differences compared with the respective controls (P and S), expressing a slight increase in antioxidant activity. On the other hand, PAW and SAW did not show any statistical significance when compared to P and S, respectively. Finally, when the samples from the two distinct beer styles were compared, once again, the darker beer exhibited a higher antioxidant activity when compared to the pale ales with the same corresponding treatment concerning wood addition.

**Figure 2:**
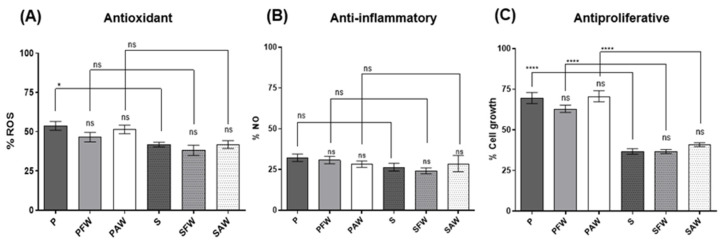
Cellular antioxidant, anti-inflammatory, and antiproliferative activities assessed through the determination of intracellular reactive oxygen species (ROS) levels in beer samples vs. LPS stimulated control (**A**); the determination of extracellular NO levels in cells exposed to beer vs. LPS control (**B**); and cell viability measured after exposure of cells to 20% sample (beer in CM) vs. medium control after 48 h exposure (**C**). ns: not significant. **** Significant at *p* < 0.001. * Significant at *p* < 0.05.

The DPPH assay results (Figure 2B), which are expressed in mg of Trolox equivalents per litre (mg TE·L^−1^) of beer sample, varied between 333.10 ± 12.72 and 382.90 ± 19.21 mg TE·L^−1^ for the pale ale style, and between 573.50 ± 55.98 and 682.70 ± 55.92 mg TE·L^−1^ for the stout style. Both pale ale and stout beers did not display any significant differences between the wood-aged samples and the respective controls (P and S). However, when compared to pale ale beer, stout beer exhibited a higher antioxidant activity. These results go along with the ones obtained in the FRAP and the overall phenolic composition assays (TPC and TFC) since phenolic compounds are heavily associated with antioxidant activity.

### 3.3. Cellular Antioxidant, Anti-Inflammatory, and Antiproliferative Activities

Cellular bioactivity was measured by using RAW 264.7 murine macrophages after LPS stimulation (intracellular ROS and extracellular NO production) and in Caco-2 human colon adenocarcinoma proliferation (antiproliferative assay). In the cellular antioxidant activity assay, all samples presented significant effects in the reduction in ROS production (ca. 50%) when compared to the LPS-stimulated control (Figure 2A). The inhibition of ROS production ranged from 46.29 ± 2.85 to 53.50 ± 3.03% for the pale and from 58.19 ± 1.52 to 61.86 ± 3.26% for the dark beer samples. In general, no significant differences were observed between the samples of the same style with and without the addition of wood chips or amid samples with different malts. An exception was the S beer, to which a slight reduction in activity was noticed when it was compared to the P sample.

Regarding the cellular anti-inflammatory assay measured by the amount of NO released into the extracellular medium, all beer samples presented activity (>60% reduction) when compared to the LPS-stimulated control (Figure 2B). The inhibition of NO production ranged between 67.74 ± 2.25–71.70 ± 1.96% for the pale and 71.34 ± 4.99%–75.75 ± 1.83% for the dark beer samples. No statistical differences were observed by comparing the beers produced with different malts or between the samples aged with and without wood.

For the antiproliferative effect, we first evaluated the IC_50_ values (% beer in CM which inhibited 50% cell growth) inferred from a dose–response curve constructed from 0.1 to 50% beer in CM. Values were determined as 18.86 ± 1.27 and 20.81 ± 1.16% for the P and S beers, respectively. The effects of wood addition were then assessed by comparing the antiproliferative activity of all samples at the average IC_50_ determined, which corresponds roughly to 20%. This approach enabled us to comparably evaluate a high number of samples and an appropriate number of replicates. The results are presented in Figure 2C. For the pale beers, the values concerning the inhibition of cell growth ranged from 29.36 to 37.05% with no statistically significant differences when the two samples with wood treatment (PFW and PAW) were compared with the control (P). For the stout style, the results ranged from 59.14 to 63.31% of inhibition of cell growth. A noteworthy difference between pale ale and stout beers was encountered, with the stout beer samples demonstrating superior antiproliferative activity with a maximum inhibition of 63.31% of cell growth for the beer treated with French oak chips, in addition to other bioactivity assays, in which the stout beer exhibited a significantly higher effect.

### 3.4. Correlation of Phenolic Composition and Bioactivity with the Type of Malt and Wood Origin

**Figure 3:**
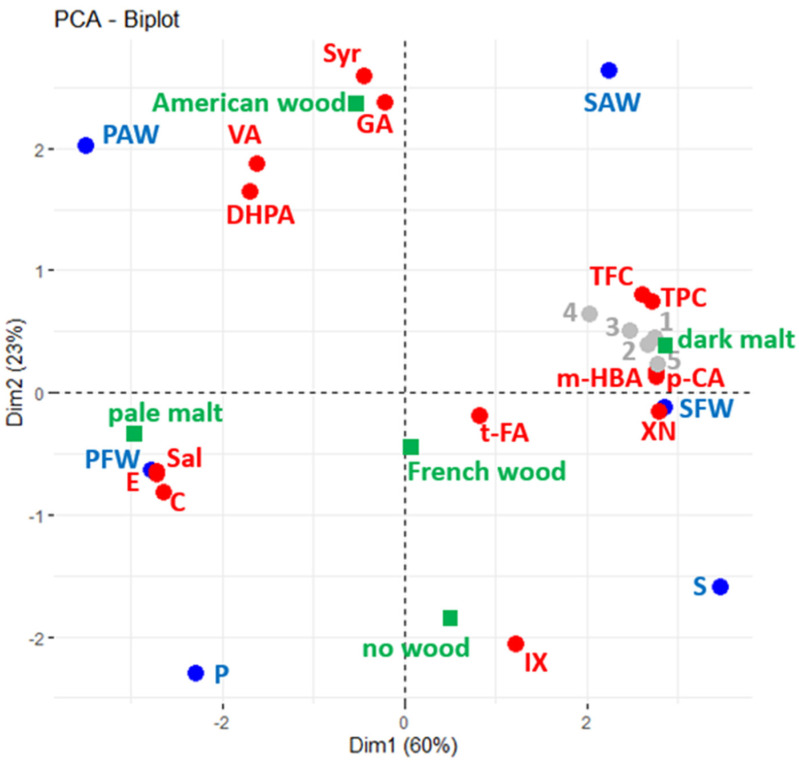
Principal Component Analysis (PCA) biplots of polyphenol composition (red) of beers (blue) used as active variables. Data from bioactivity assays (grey) used as passive supplementary variables (1 = FRAP, 2 = DPPH, 3 = antioxidant, 4 = anti-inflammatory, and 5 = antiproliferative). In green, the type of malt and wood origin are explored as supplementary factors.

To summarise and best visualise the overall relationship between the six beers and the bioactive proprieties, Principal Component Analysis (PCA) was performed using chemical composition data from TFC, TPC, and phenol quantifications (active variables—red spots) of studied beers plotted in blue (Figure 3). Results from bioactivity assays are shown as supplementary variables, which are displayed as a layer over the initial plotted correlation (grey spots). The type of malt and wood origin were explored as supplementary factors (in green).

The first two dimensions of the PCA explained 83% of data variance. According to the squared cosine (Supplementary Table S1), all beers and most of the variables are explained in these two dimensions (only the quantification of trans-ferulic acid is best explained in the third dimension).

By taking into consideration the squared cosine and Pearson correlation coefficients (Supplementary Table S2), it was possible to denote that the five bioactive properties evaluated correlate to the malt profile, as the values were higher in dark beers with higher amounts of TPC, TFC, m-hydroxybenzoic acid, p-coumaric acid, and xanthohumol. On the other hand, (+)-catechin, (−)-epicatechin, and salicylic acid were more prominent in the pale ale beers.

Moreover, it is also observed that some compounds presented more connection with the wood origin, such as the gallic acid, syringic acid, and vanillic acid amounts that are associated with American wood.

## 4. Discussion

Concentrations found in the analysed beers were in accordance with mean values and type of compounds found in the studies reporting the phenolic composition of beers [18,19,20,21] and in the beer flavour database (ASBC) regarding the contribution of some phenolics to the beer flavour. Malt phenolics contribute to 70–80% of total polyphenols in beer, with darker malts being richer in phenolics from the kilning and roasting process [20]. The higher values of phenolic compounds in the S beers, which contain dark malts, agree with previous reports, which demonstrated that the darker the malt, the greater the content of phenolic compounds released into the beer in the brewing process. Regarding catechin values, a previous study on the phenolic composition of craft beers describes the same pattern noticed in our results. The authors characterised a Pilsen beer (comprising exclusively light malt) containing 108.3 mg/L of catechin, a higher concentration than the dark beers, the amount of which ranged from 16.8 to 65.2 mg/L [22]. Other than the type of malt, phenolic compositions appeared to be equally associated with the wood origin. French wood chips, in general, are considered richer in phenolic compounds than the wood of American oaks [23,24,25]. This pattern transferred to the extracting beverage, which, in most previous studies, is wine. However, in the present study, beers treated with French oak presented similar or even lower phenolic composition. Although distinct from results of previous studies related to phenolics transfer from wood to wine, our results follow the same trend described in beer by other authors who evaluated the influence of wood ageing on monophenol concentrations of beers and demonstrated that its phenolic composition was more associated with American than French oak chips [26].

The antioxidant activity results share the same propensity manifested in the total flavonoid and phenolic compositions: dark beers exhibited the highest antioxidant activities. The FRAP assay results were higher than those reported by Piazzon et al. [27], whose values ranged from 329 to 700 mg FSE·L^−1^. The obtained results in DPPH assays are, much like the ones obtained for the TPC assay, superior to previously reported values in the literature, which range from 60 to 338 mg TE·L^−1^ for commercial beers [12,28]. The differences may be justified by the lack of the filtration process of samples used in this work, which increased the available amount of phenolic antioxidant compounds which were otherwise absent (the filtration process can remove a significant portion of antioxidant phenolic compounds).

With respect to the cellular anti-inflammatory activity, our results are, in part, in accordance with previous studies. Kanuri et al. (2015) [29] reported that Pilsner beer tested in RAW 264.7 cells, which were stimulated with LPS, managed to attenuate the LPS effect in iNOS and TNF-α mRNA expression, thereby exerting a significant anti-inflammatory activity, concurring with the obtained results for the tested beer samples in this work. However, the authors also reported that the stout style (dark beer) failed to display any activity compared to the LPS control, contradicting the verified tendency in this work. Notwithstanding, it was also previously reported that ferulic acid can diminish NO production in RAW 264.7 cells [30]. This finding agrees with our results since we were able to quantify *t*-ferulic acid in both pale and dark beers in similar amounts (Table 2). We verified herein that the wood-ageing process, in the conditions tested, overall did not accentuate bioactivities for the beers in Raw 264.7 and Caco-2 cell assays. In all cellular bioactive assays, no statistical differences were observed when the wood-aged beers were compared with the respective controls. As previously reported [31], and herein verified by the high bioactivity of the controls without wood addition, the fundamental beer ingredients (hops and malts) strongly contribute to the beer matrix, which itself contains potent bioactive substances able to promote cellular bioactivities. In the PCA graph, although it was possible to observe that the phenolic composition, at the individual compound level, is correlated to both the type of malt and wood origin, the bioactivity of the beers is exclusively associated with the malt, which is probably related to the well-described bioactivity from TFC and TPC [32,33] and xanthohumol [34] contents. Notwithstanding the reported results, it is important to note that the physiological effects of ethanol in beers were not accounted for herein. However, in recent years, there has been an increased interest in reducing the alcohol content in the beverage, thus making it possible to obtain the beneficial effects of beer without the alcoholic toxicity. In addition, the bioactive effects found can be much more complex in the human body, which open ways for its exploitation concerning the fate of the molecules in the gastrointestinal digestion, its metabolism, and its interactions.

## 5. Conclusions

The present work assessed the effects of the wood-ageing process on beer bioactivity and phenolic composition. Thirteen individual phenolic compounds were identified and quantified in the six beers studied, with concentration values that were corroborated by the available literature for most of them. The beer samples aged with the American oak wood chips exhibited the more promising results for phenolic extraction, namely for compounds such as gallic, 3,4-dihydroxyphenylcetic, vanillic, and syringic acids. Overall, phenolics composition was more differentiated in pale beers when samples were compared with and without wood treatments.

Chemical antioxidant activities measured via FRAP and DPPH were noticeably higher in dark beers, and it was noticed that the wood addition resulted in an incremental change only in the case of FRAP when French wood was used. Overall, beer samples were found to possess a noteworthy antioxidant activity and higher phenolic and flavonoid concentrations when compared to the literature, which refers mostly to commercial beers.

Antioxidant, anti-inflammatory, and antiproliferative activities of beer aged with oak wood chips were assessed via cell culture assays. No significant differences were found between pale and dark beers whether samples were matured or not in wood. The exception was the antiproliferative activity that was found to be significantly higher for the dark beer accompanying the different profiles of the chemical assays.

This work reported valuable information regarding craft beer phenolic and flavonoid concentrations, as well as the chemical and cellular bioactivity of non-filtered craft beers, for which information is relatively scarce in the literature.

From the present results, it could be important to further exploit the effects of the quantity, time of exposure, and toast degree of the oak wood chips and their respective impact on the amount of extractable phenolic compounds and expected global bioactivity augmentation. Particularly interesting would be to verify if the increment in beer–wood contact time could result in more phenolics transfer with a consequent increase in bioactivity.

## Figures and Tables

**Table 1:** Sample codes of beer produced and respective malt profiles and maturation conditions.

Code	Malt Profile	Maturation
P	100% of pale ale malt	without wood
PFW		French oak
PAW		American oak
S	90% of pale ale malt 10% of dark malt (8% chocolate + 2% black)	without wood
SFW		French oak
SAW		American oak

**Table 2:** Phenolics quantitative composition of the beer with and without maturation in American and French oak chips.

	Pale Beer			Dark Beer			
	P	PFW	PAW	S	SFW	SAW	*p*-Values
TPC	772.0 ±92.6 ^a^	810.1 ± 106.8 ^ab^	950.4 (574.1–1795.2) ^b^	1412.1 (877.1–2435.9) ^c^	1509.5 ± 220.5 ^c^	1540.3 ± 311.2 ^c^	<0.001 ^kD^
TFC	60.3 (55.0–62.9) ^ab^	54.1 ± 11.1 ^a^	54.1 ± 8.6 ^a^	83.5 ± 12.7 ^bc^	91.8 ± 19.1 ^c^	100.2 ± 12.2 ^c^	<0.001 ^kD^
GA	n.d.	0.244 ± 0.026	0.269 (0.169–0.270)	n.d.	0.226 ± 0.031	0.309 ± 0.018	0.082 ^kD^
DHPA	0.052 ± 0.001 ^abc^	0.043 ± 0.003 ^ab^	0.124 ± 0.032 ^c^	0.030 (0.030–0.049) ^a^	0.039 (0.028–0.039) ^a^	0.057 ± 0.005 ^bc^	0.009 ^kD^
*m*-HBA	0.104 ± 0.004 ^ab^	0.091 ± 0.007 ^a^	0.102 ± 0.011 ^ab^	0.166 ± 0.030 ^c^	0.150 ± 0.026 ^bc^	0.149 ± 0.011 ^bc^	0.001 ^AT^
VA	0.436 ± 0.008 ^a^	0.400 ± 0.038 ^a^	0.706 ± 0.106 ^b^	0.360 ± 0.079 ^a^	0.341 ± 0.066 ^a^	0.503 ± 0.052 ^a^	<0.001 ^AT^
C	1.139 ± 0.100 ^b^	0.938 ± 0.088 ^b^	0.976 ± 0.120 ^b^	0.498 ± 0.047 ^a^	0.511 ± 0.098 ^a^	0.480 ± 0.030 ^a^	<0.001 ^AT^
Syr	n.d.	0.020 ± 0.002 ^a^	0.198 ± 0.030 ^b^	0.060 ± 0.014 ^a^	0.062 ± 0.016 ^a^	0.195 ± 0.024 ^b^	<0.001 ^AT^
Van	n.q.	n.q.	n.q.	n.q.	n.q.	n.q.	
E	0.353 ± 0.016 ^b^	0.288 ± 0.021 ^a^	0.294 ± 0.019 ^a^	n.d.	n.d.	n.d.	0.011 ^AT^
*p*-CA	0.050 ± 0.006 ^a^	0.029 ± 0.009 ^a^	0.033 ± 0.006 ^a^	0.099 ± 0.019 ^b^	0.090 ± 0.019 ^b^	0.095 ± 0.006 ^b^	<0.001 ^AT^
Sal	0.688 ± 0.031 ^c^	0.553 ± 0.039 ^b^	0.574 ± 0.041 ^b^	0.104 ± 0.032 ^a^	0.090 ± 0.026 ^a^	0.127 ± 0.010 ^a^	<0.001 ^AT^
*t*-FA	0.408 ± 0.011	0.282 ± 0.046	0.359 ± 0.047	0.384 ± 0.061	0.344 ± 0.066	0.386 ± 0.024	0.072 ^AT^
IX	0.458 ± 0.079 ^ab^	0.366 ± 0.028 ^a^	0.371 (0.370–0.416) ^ab^	0.524 ± 0.088 ^b^	0.403 (0.402–0.517) ^ab^	0.352 ± 0.023 ^a^	0.040 ^kD^
XN	0.488 ± 0.223 ^a^	0.374 ± 0.028 ^a^	0.373 ± 0.036 ^a^	1.248 ± 0.137 ^b^	1.079 ± 0.245 ^b^	0.955 ± 0.035 ^b^	<0.001 ^AW^

Total phenolic contents (TPC) are expressed as mg of gallic acid equivalents per litre (mg GAE/L); total flavonoid contents (TFC) are expressed in mg of (+)-catechin equivalents per litre (mg CE/L). Results of phenolic compounds are expressed as mg/L. Data modelled via normal distribution (expressed as mean ± standard deviation) were evaluated via one-way ANOVA/Tukey ^(AT)^ or Welch ^(AW)^ test since homogeneity of variances was, or was not, respectively, confirmed via Levene’s test. Kruskal–Wallis chi-squared/Dunn ^(kD)^ test were performed for data without normal distribution, expressed as median (minimum–maximum). Different letters in a row show statistically significant differences at *p*-values between means and medians. P—pale beer, PFW—pale beer with French wood, PAW—pale beer with American wood, S—dark beer, SFW—dark beer with French wood, SAW—dark beer with American wood, GA—gallic acid, DHPA—3,4-dihydroxyphenylacetic acid, *m*-HBA—*m*-hydroxybenzoic acid, VA—vanillic acid, C—(+)-catechin, Syr—syringic acid, Van—vanillin, E—(−)-epicatechin, *p*-CA—*p*-coumaric acid, Sal—salicylic acid, *t*-FA—*trans*-ferulic acid, IX—isoxanthohumol, XN—xanthohumol. n.d.—not detected, n.q.—not quantified.

## Data Availability

Data is contained within the article or Supplementary Material.

## Supplementary Materials

The following supporting information can be downloaded at: https://www.mdpi.com/article/10.3390/foods12061237/s1, Table S1: Squared cosines of all five dimensions of compounds (variables) and beers (observations), obtained via Principal Component Analysis (PCA) of GC-O results. Table S2: Pearson correlation.
