# Supplementary material for: Bioactive Properties and Phenolic Composition of Wood-Aged Beers: Influence of Oak Origin and the Use of Pale and Dark Malts
Source: Foods. 2023 Mar 14;12(6):1237. doi: 10.3390/foods12061237 (PMC10048385; doi:10.3390/foods12061237)
Supplement: Supplementary file 1 [file foods-12-01237-s001.zip › foods-2246243-supplementary.pdf]

Supplementary Table S1. Squared cosines of the all five dimensions of compounds (variables) and beers (observations), obtained by Principal Component Analysis (PCA).

	**Dim 1**	**Dim 2**	**Dim 3**	**Dim 4**	**Dim 5**
Variance	8.399	3.215	1.864	0.471	0.05
Percentage of variance	59.99%	22.97%	13.31%	3.37%	0.36%
Cumulative % of variance	59.991	82.958	96.273	99.64	100
Active variables					
TFC	**0.854435**	0.079129	0.000118	0.061569	0.004748
TPC	**0.922280**	0.068140	0.000333	0.007845	0.001402
GA	0.006063	**0.705755**	0.270982	0.003571	0.013629
DHPA	**0.360124**	0.339452	0.207212	0.085410	0.007802
m.HBA	**0.957332**	0.003541	0.031928	0.006633	0.000566
VA	0.330624	**0.440891**	0.205278	0.023010	0.000197
C	**0.877322**	0.084845	0.019712	0.013721	0.004400
Syr	0.025188	**0.841591**	0.117535	0.002979	0.012708
E	**0.930184**	0.056599	0.006446	0.006603	0.000167
p.CA	**0.960891**	0.002134	0.024459	0.012515	0.000002
Sal	**0.920564**	0.051808	0.012918	0.014701	0.000009
t-FA	0.085035	0.004878	**0.778718**	0.129634	0.001735
IX	0.188204	**0.533470**	0.180532	0.094748	0.003046
XN	**0.980536**	0.003108	0.007951	0.008370	0.000035
Passive variable					
DPPH	**0.892884**	0.019192	0.000410	0.000729	0.086785
FRAP	**0.942940**	0.024762	0.015193	0.005225	0.011881
Caco-2 Antiproliferative	**0.965272**	0.006401	0.025010	0.003188	0.000130
RAW 264.7 Anti-inflammatory	**0.519958**	0.051620	0.004767	0.269022	0.154633
RAW 264.7 Antioxidant	**0.764467**	0.031552	0.180699	0.018683	0.004599
Observations					
Pale malt No-Wood	0.404108	**0.408397**	0.125916	0.060937	0.000643
Pale malt French Oak	**0.541606**	0.028484	0.426805	0.000690	0.002415
Pale malt US Oak	**0.662556**	0.221250	0.081812	0.034026	0.000356
Dark malt No-Wood	**0.752211**	0.157577	0.053099	0.033381	0.003732
Dark malt French Oak	**0.859573**	0.001729	0.117393	0.003363	0.017942
Dark malt US Oak	0.391060	**0.541748**	0.000581	0.064751	0.001861

For each variable and observation, values in bold correspond to the dimension that the squared cosine is the largest.

Supplementary Table S2. Pearson correlation

	**r**	***p*-Value**
Correlation with Dim 1		
XN	0.990	0.0001
Caco.2.antiproliferative	0.982	0.0005
p.CA	0.980	0.0006
m.HBA	0.978	0.0007
FRAP	0.971	0.0012
TPC	0.960	0.0023
DPPH	0.945	0.0045
TFC	0.924	0.0084
RAW.264.7.Antioxidant	0.874	0.0227
C	-0.937	0.0059
Sal	-0.959	0.0024
E	-0.964	0.0019
Type of malt	0.970	0.0003
Correlation with Dim 2		
Syr	0.917	0.0100
GA	0.840	0.0363
Wood origin	0.970	0.0051
Correlation with Dim 3		
t-FA	0.917	0.0100
